# Supplementary material for: Effects of Benzalkonium Chloride Contents on Structures, Properties, and Ultrafiltration Performances of Chitosan-Based Nanocomposite Membranes
Source: Membranes (Basel). 2022 Feb 25;12(3):268. doi: 10.3390/membranes12030268 (PMC8952018; doi:10.3390/membranes12030268)
Supplement: Supplementary file 1 [file membranes-12-00268-s001.zip › membranes-1578994-supplementary.pdf]

membranes

Supplementary Materials

Effects of Benzalkonium Chloride Contents on Structures, Properties, and Ultrafiltration Performances of Chitosan-Based Nanocomposite Membranes

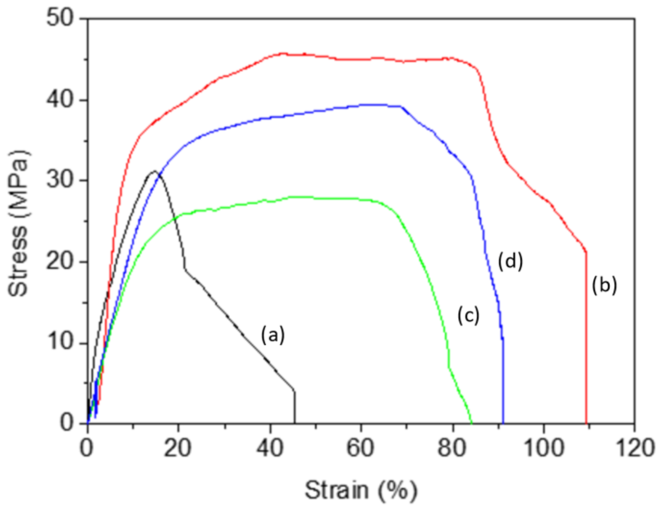

(I)

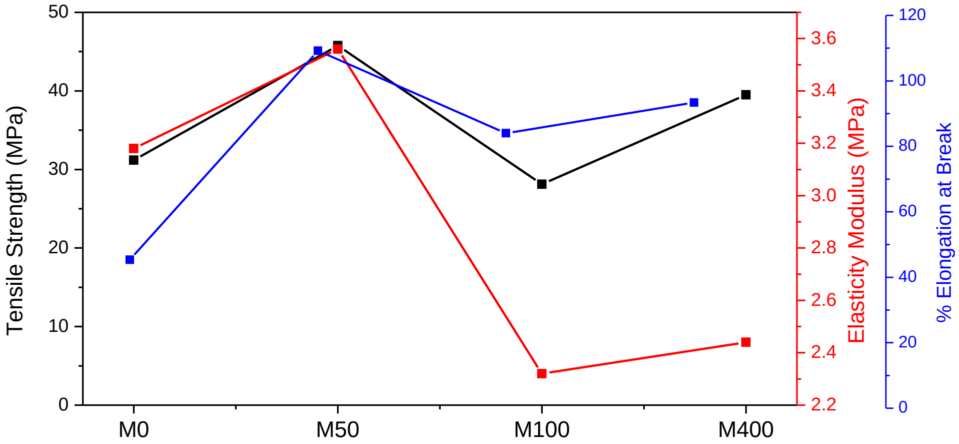

(II)

Figure S1. (**I**) Stress vs. strain curves of (a) M0, (b) M50, (c) M100 and (d) M400; (**II**) Tensile strength, Young's modulus, and elongation at break of nanocomposite membranes.

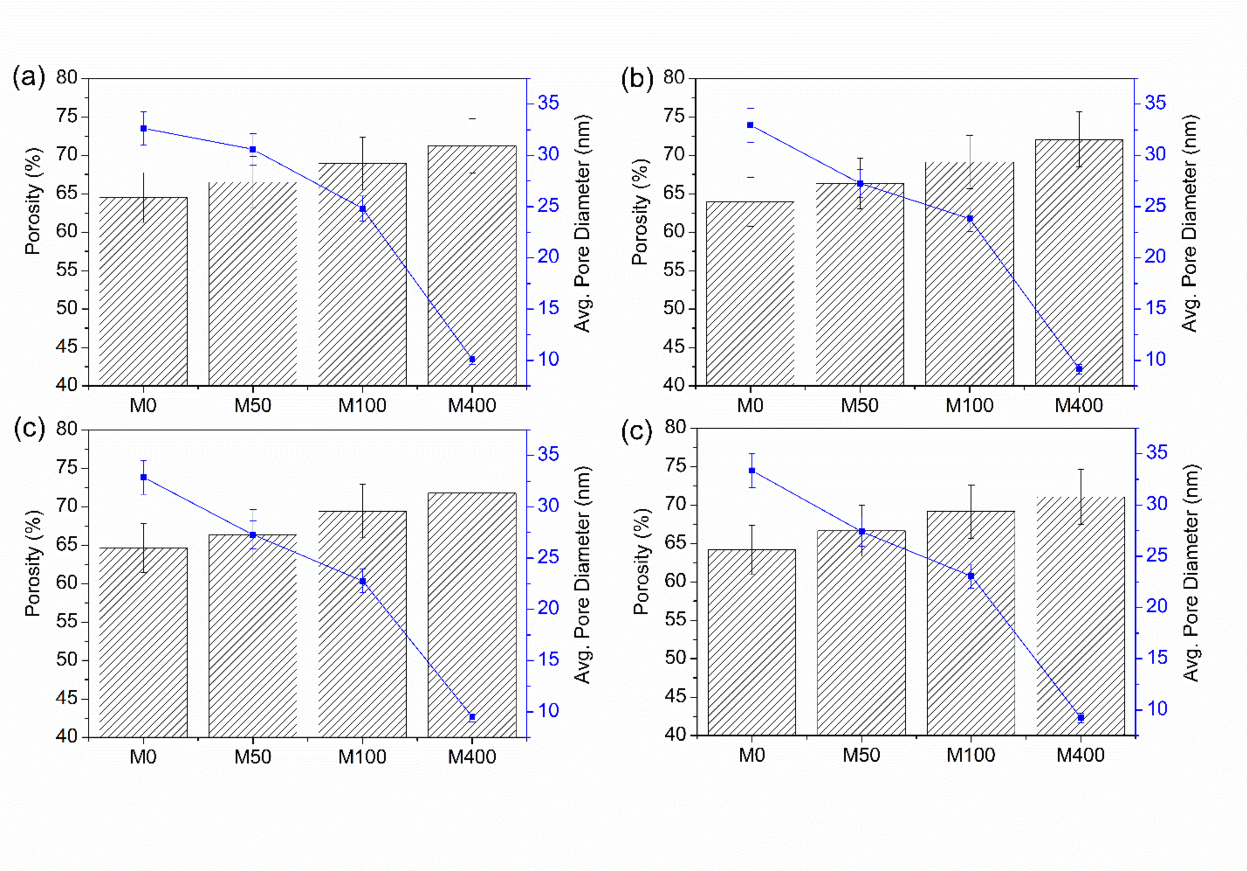

Figure S2. Porosity and average pore size of nanocomposite membranes with different BKC contents and thicknesses: (**a**) 0.05 mm, (**b**) 0.06 mm, (**c**) 0.07 mm and (**d**) 0.08 mm.

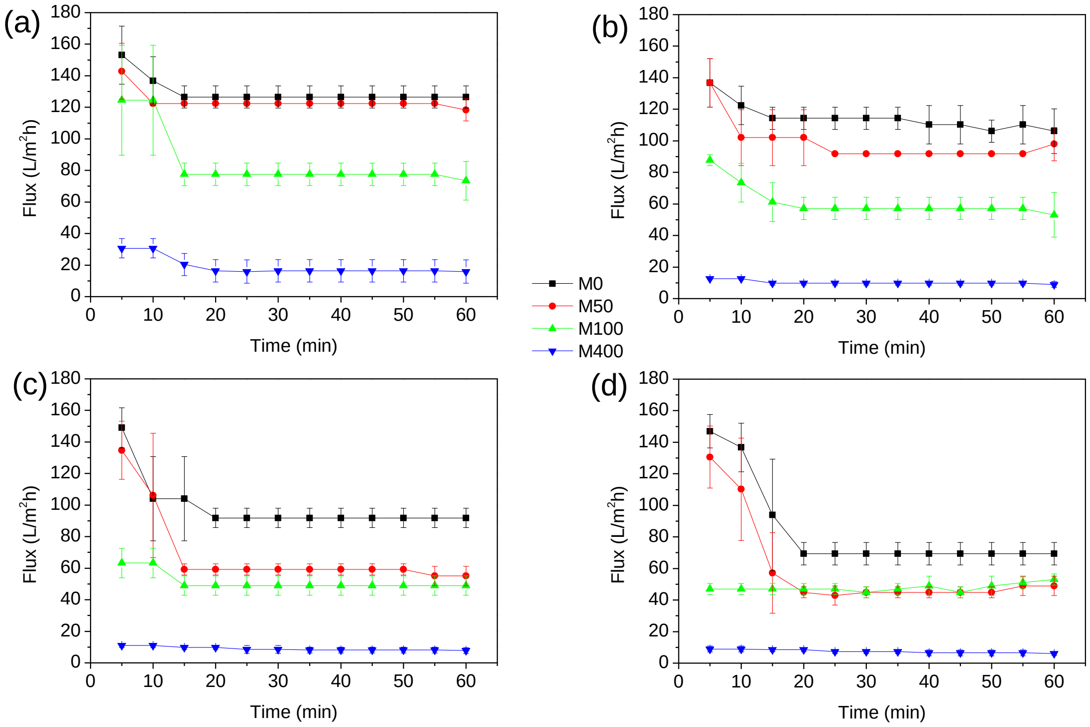

Figure S3. Flux-time relationship for various nanocomposite membranes at various thicknesses for the dead-end filtration model: (**a**) 0.05 mm, (**b**) 0.06 mm, (**c**) 0.07 mm and (**d**) 0.08 mm.

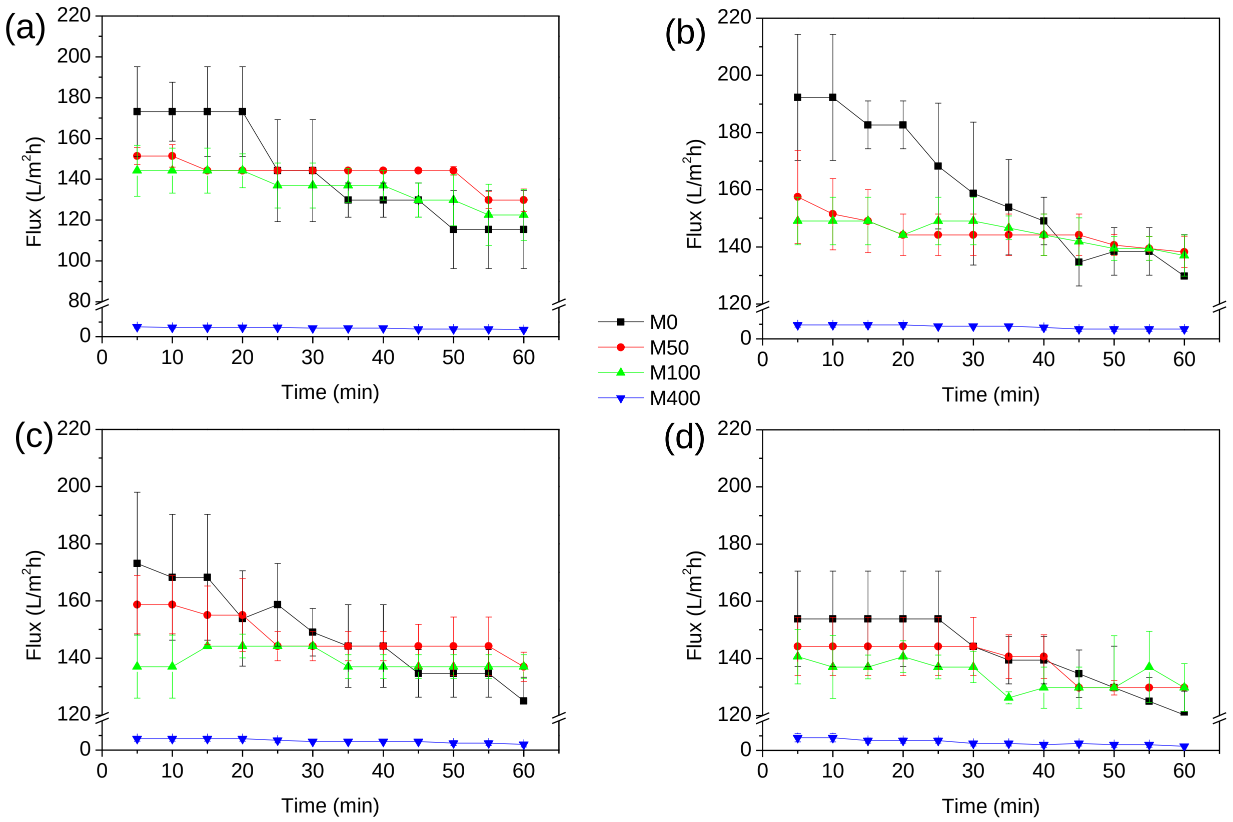

Figure S4. Flux-time relationship for various nanocomposite membranes at various thicknesses for the crossflow filtration model: (**a**) 0.05 mm, (**b**) 0.06 mm, (**c**) 0.07 mm and (**d**) 0.08 mm.

Table S1. Comparisons of the performance of similar membranes.

No.	Precursor	Pressure (Bar)	Pure Water Flux L/m² h	Rejection	Source
1	CS/PEG/BKC /CNT	4	129.59	40 (BSA)	This work
2	CS/PVA/MMT	1	25.72	88.34 (Chromium)	Sangeetha, K., Sudha, P. N., & Sukumaran, A. (2019). Novel chitosan based thin sheet nanofiltration membrane for rejection of heavy metal chromium. *International journal of biological macromolecules*, *132*, 939-953.
3	CS/GO	4	10	95 (BSA)	Liu, T., Graham, N., & Yu, W. (2021). Evaluation of a Novel Composite Chitosan–Graphene Oxide Membrane for NOM Removal during Water Treatment. *Journal of Environmental Chemical Engineering*, 105716.
4	MWCNT/CS	3	5.73	65 (NaCl)	Alshahrani, A. A., Algamdi, M. S., Alsohaimi, I. H., Nghiem, L. D., Tu, K. L., Al-Rawajfeh, A. E., & in het Panhuis, M. (2020). The rejection of mono-and di-valent ions from aquatic environment by MWNT/chitosan buckypaper composite membranes: Influences of chitosan concentrations. *Separation and Purification Technology*, *234*, 116088.
